# Multiple Cancer/Testis Antigens Are Preferentially Expressed in Hormone-Receptor Negative and High-Grade Breast Cancers

**DOI:** 10.1371/journal.pone.0017876

**Published:** 2011-03-18

**Authors:** Yao-Tseng Chen, Dara S. Ross, Rita Chiu, Xi K. Zhou, Yunn-Yi Chen, Peishan Lee, Syed A. Hoda, Andrew J. Simpson, Lloyd J. Old, Otavia Caballero, A. Munro Neville

**Affiliations:** 1 Department of Pathology and Laboratory Medicine, Weill Medical College of Cornell University, New York, New York, United States of America; 2 Ludwig Institute for Cancer Research, New York Branch, New York, New York, United States of America; 3 Department of Public Health, Weill Medical College of Cornell University, New York, New York, United States of America; 4 Department of Pathology, University of California San Francisco, San Francisco, California, United States of America; Sun Yat-sen University Medical School, China

## Abstract

**Background:**

Cancer/testis (CT) antigens are protein antigens normally expressed only in germ cells of testis, and yet are expressed in a proportion of a wide variety of human cancers. CT antigens can elicit spontaneous immune responses in cancer patients with CT-positive cancers, and CT antigen-based therapeutic cancer vaccine trials are ongoing for “CT-rich” tumors. Although some previous studies found breast cancer to be “CT-poor”, our recent analysis identified increased CT mRNA transcripts in the ER-negative subset of breast cancer.

**Methodology/Principal Findings:**

In this study, we performed a comprehensive immunohistochemical study to investigate the protein expression of eight CT genes in 454 invasive ductal carcinomas, including 225 ER/PR/HER2-negative (triple-negative) carcinomas. We found significantly more frequent expression of all eight CT antigens in ER-negative cancers, and five of them—MAGEA, CT7, NY-ESO-1, CT10 and CT45, were expressed in 12–24% of ER-negative cancers, versus 2–6% of ER-positive cancers (*p*<0.001 to 0.003). In comparison, GAGE, SAGE1 and NXF2 were only expressed in 3–5% of ER-negative and 0–2% of ER-positive cancers. ER-negative cancers were also more likely to simultaneously co-express multiple CT antigens, with 27% (34/125) of ER-negative, CT-positive tumors expressing three or more CT antigens. HER2 status had no consistent effect on CT expression, and triple-negative carcinomas showed similar frequencies of MAGEA and NY-ESO-1 expression as ER-negative/HER2-positive carcinomas. More frequent CT expression was also found in tumors with higher nuclear grade (*p*<0.001 to p = 0.01) and larger in size (>2 cm).

**Conclusions/Significance:**

CT antigens are preferentially expressed in hormone receptor-negative and high-grade breast cancer. Considering the limited treatment options for ER/PR/HER2 triple-negative breast cancer, the potential of CT-based immunotherapy should be explored.

## Introduction

Cancer/testis (CT) antigens are protein antigens that are normally expressed in the germ cells of adult testis and developing fetal testis and ovary, but not in any other adult tissues. Examination of various types of human cancer showed CT gene activation and protein expression in a proportion of human cancers in a lineage-unrelated fashion [1], [2], [3], [4]. Due to this restricted pattern of expression, CT antigens are often recognized by the immune system of cancer patients, and this spontaneous immunogenicity raises the possibility of their use as therapeutic cancer vaccine targets. The prototype examples of CT antigens, MAGE-A [5] and NY-ESO-1 [6], were among the first human tumor antigens shown to elicit a spontaneous cytotoxic T cell response in cancer patients[5], [7]. Cancer vaccine trials with these two antigens have demonstrated their capability of inducing humoral and cell-mediated immune responses in some patients, and examples of clinical responses have also been documented [7], [8], [9], [10].

One practical consideration that would determine the potential utility of CT-based cancer vaccine is the frequency of CT antigen expression in the specific tumor type being considered, and cancers of different tissue origin have been shown to differ significantly in this aspect. Melanoma, ovarian cancer, lung cancer and bladder cancer are examples of “CT-rich” tumors, whereas renal cancer, colorectal cancer and lymphoma/leukemia are “CT-poor”, rarely expressing CT antigens [4]. Relatively few studies have evaluated CT expression in breast cancer, most of them focusing on the expression of NY-ESO-1 and MAGEA family [11], [12], [13], [14], [15]. The data from these studies were highly variable, with the reported NY-ESO-1 positive rate between 2.1% to 40% in different immunohistochemical studies and MAGE-A positive rate between <20% to 74%. The reason for this wide variation is not entirely clear but may partially be explained by the different patient populations that were examined (see Discussion).

For a given tumor type, the frequency of CT expression is often dependent upon tumor grade, stage, and histological types. Tumors of higher grade–e.g. in bladder cancer [16]–and at more advanced stage–e.g. in melanoma [17] –, more frequently expressed CT antigens than low grade or early stage tumors. In lung cancer, squamous cell carcinomas and neuroendocrine carcinomas more frequently expressed CT antigens than adenocarcinomas, demonstrated at both mRNA and at the protein levels [2]. Consistent with this notion, we recently found significantly higher frequency of CT mRNA expression in estrogen receptor (ER) and progesterone receptor (PR) negative breast cancer cell lines and primary breast cancers, including MAGE-A3, MAGE-A6, NY-ESO-1, MAGE-A12, LAGE-1, CSAG2 etc [12]. Subsequent immunohistochemical analysis in a series of 153 unselected cases of breast cancer confirmed the more frequent expression of MAGE-A and NY-ESO-1 protein in ER-negative tumors, and similar findings were also observed by analyzing 19 cases of ER, PR and HER2 triple-negative breast cancer. Our goal in the present study was to expand that study and carry out a comprehensive immunohistochemical analysis of eight CT antigens in a large cohort of primary ductal breast cancer with different ER, PR and HER2 status. We found significantly higher expression rate of all eight CT antigens in the ER negative group and tumors with high nuclear grade and larger size also showed more frequent CT expression. These findings indicate that a CT antigen cancer vaccine, particularly if polyvalent, can potentially represent an important therapeutic option for patients with ER-negative breast cancer, including the clinical aggressive triple-negative subtype for which the treatment options are limited.

## Results

### Expression characteristics of individual CT antigens

**Figure 1:**
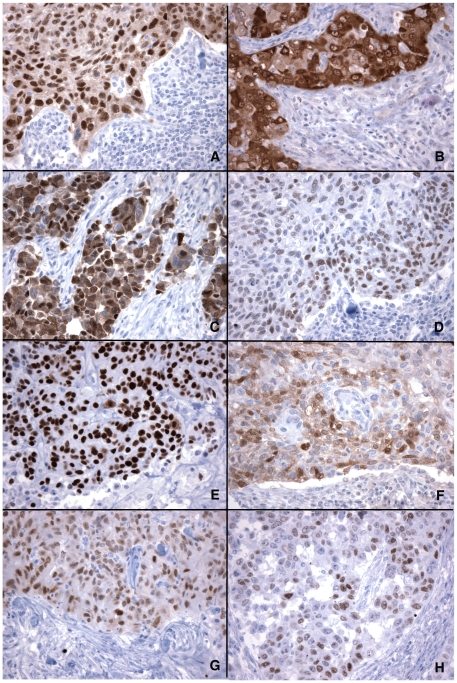
Immunohistochemical analysis of CT antigen expression in breast cancer. Eight CT antigens were analyzed– MAGEA (A), NY-ESO-1 (B), CT7 (C), CT10 (D), CT45 (E), GAGE (F), NXF2 (G) and SAGE1 (H). Of these, MAGEA, NY-ESO-1, CT7 and GAGE showed mixed nuclear and cytoplasmic staining, whereas CT10, CT45, NXF2 and SAGE1 were purely or predominantly nuclear proteins. (Magnifications: 400X).

**Figure 2:**
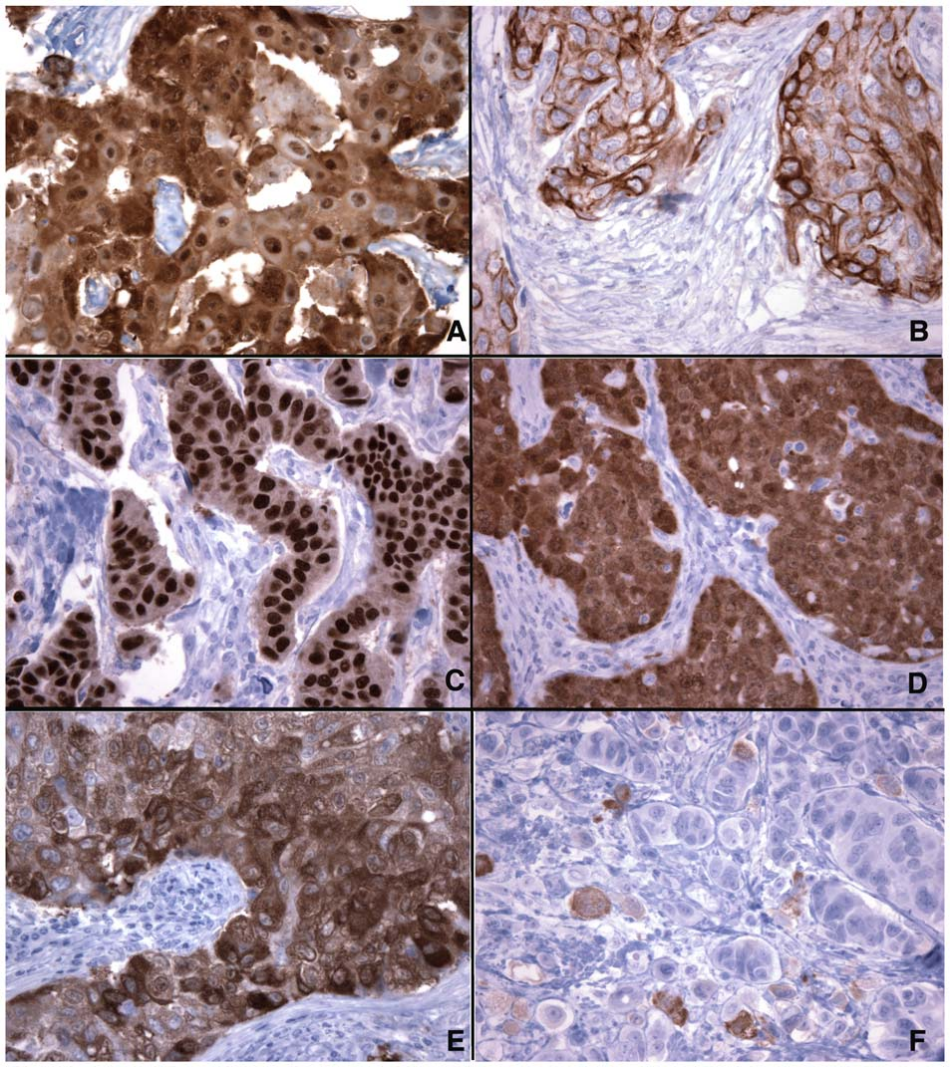
Variations in cellular and subcellular distributions of CT antigens in breast cancer. (A, B): NY-ESO-1 staining of two ER-negative carcinomas, showing mixed nuclear and cytoplasmic staining in (A) and pure cytoplasmic staining in (B). (C, D): MAGEA staining of two ER-negative carcinomas, showing predominantly nuclear (C) and cytoplasmic (D) staining, respectively. (E, F): CT7 staining in two cases, showing diffuse positivity in >90% of tumor cells in (E), as compared to (F) which showed only scattered positive cells and many tumor cells were negative. (Magnifications: 400X).

Figure 1 illustrates the typical staining patterns of the eight CT antigens in breast cancer. Similar to their subcellular localization in normal testicular germ cells, CT10, CT45, SAGE1 and NXF2 showed nuclear staining in all positive cases, and MAGEA, NY-ESO-1 and GAGE proteins are present as both nuclear and cytoplasmic proteins. The relative abundance in the nuclear and cytoplasmic compartments, however, varied significantly between individual tumors, as illustrated by NY-ESO-1 (Fig. 2A vs. 2B) and MAGE-A (Fig. 2C vs. 2D) staining. An exception was CT7, which is a cytoplasmic protein in normal germ cells but showed mixed cytoplasmic and nuclear distribution in most positive cases (Fig. 1C), with either nuclear (Fig. 1C) or cytoplasmic (Fig. 2E) compartment dominating.

**Figure 3:**
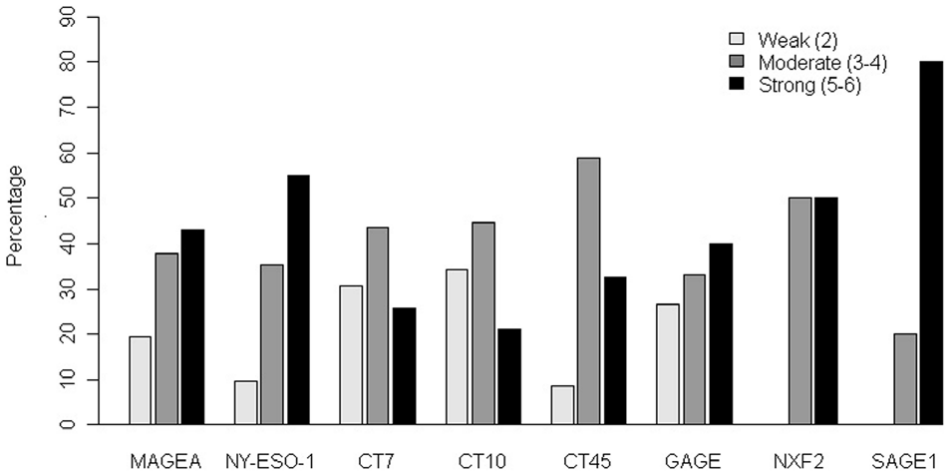
Distribution of immunohistochemical reactivity percentages among CT-positive breast cancer. The immunoreactivity in each positive case was given an extent score (1, <10% cells positive; 2, 10-50% cells positive; 3, >50% cells positive) and an intensity score (1, +; 2, ++; 3, +++). A combined score of 2 is considered weak positive, 3 to 4 as moderate, and 5 to 6 as strong positive. Most CT-positive cases showed moderate to strong reactivity, but CT7 and CT10 had more weak positive cases, see text.

The extent and intensity of CT expression in each positive case was given a combined numerical score (between 2 to 6, see Materials and Methods), and the distribution of the scores for individual CT antigen is shown in Figure 3. The majority of cases positive for MAGEA, NY-ESO-1, CT45, GAGE, NXF2 or SAGE1 showed moderate or strong expression, and <30% of positive cases showed weak and very focal staining for these CT antigens. In comparison, CT7 or CT10 positive cases had higher percentages of weaker positive cases, and 30–35% showed only weak and very focal (<10%) staining of the tumor cells, as exemplified in Fig. 2F for CT7. The weaker staining of CT10 in tumor, however, might be partially due to the lower antibody strength of anti-CT10 antibody, as CT10 staining in spermatogonia was also weaker than other nuclear CT antigens (data not shown). No statistical significant difference was observed in the extent/intensity distributions of CT expression between carcinomas of different ER status, HER2 status, or other pathological parameters such as tumor size, nuclear grade or lymph node status (see below).

### Frequency of CT antigen expression

Different CT antigens were expressed at significantly different frequencies in breast cancer, and this was observed in both Cornell series and UCSF series (Table 1). Combining both Cornell and UCSF series, MAGE-A showed most frequent CT expression (77/454, 17.0%), followed by CT7 (13.7%), NY-ESO-1 (11.2%), CT45 (10.1%) and CT10 (8.4%). However, since both cohorts were designed to enrich for ER-negative breast cancers, these numbers do not represent the expression frequencies in an unselected breast cancer population. The remaining three CT antigens–GAGE, SAGE1 and NXF2, were infrequently or rarely expressed, positive only in 3.5%, 2.2% and 1.8% of the series, respectively. These three CT antigens were not included in later comparisons.

**Table 1:** Frequency of CT antigen expression in invasive ductal carcinoma of the breast.

	MAGEA	NY-ESO-1	CT7	CT10	CT45	GAGE	NXF2	SAGE1
Cornell	48/289(16.7%)	25/289(8.7%)	44/289(15.2%)	25/289(8.7%)	26/289(9.0%)	11/289(3.8%)	6/289(2.1%)	6/289(2.1%)
UCSF	29/165(17.6%)	26/165(15.8%)	18/165(10.9%)	13/165(7.9%)	20/165(12.1%)	5/165(3.0%)	2/165(1.2%)	4/165(2.4%)
Total (No.)	77/454(17.0%)	51/454(11.2%)	62/454(13.7%)	38/454(8.4%)	46/454(10.1%)	16/454(3.5%)	8/454(1.8%)	10/454(2.2%)

### ER-positive versus ER-negative tumors

**Figure 4:**
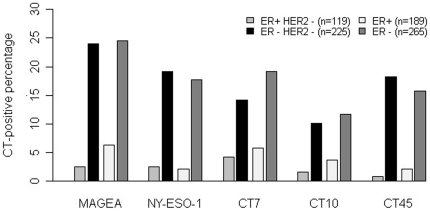
Distribution of CT antigen expression percentages in ER-positive and ER-negative breast cancer. Comparisons were carried out for each CT antigen between ER+HER2- and ER-HER2- cases and between all ER+ and ER- cases. Significant differences were found for all comparisons (P<0.005).

To exclude the possible influence of HER2, the correlation between ER status and CT expression was first compared using the 119 ER+HER2- and 225 ER-HER2- (triple-negative) cases in both Cornell and UCSF cohorts (Figure 4). All five main CT antigens showed higher expression frequency in the ER-negative than in the ER-positive group, with statistically significant differences for all comparisons (*p*<0.001 for MAGEA, NY-ESO-1, and CT45, and *p*<0.005 for CT7, and CT10). Comparison of all 189 ER+ and 265 ER- tumors irrespective of their HER2 status led to the same finding (p = 0.003 for CT10 and p<0.001 for all other CTs), as was the comparison between the 225 triple-negative cases and the 189 ER+ cases (p = 0.013 for CT10, 0.006 for CT7 and <0.001 for MAGE-A, NY-ESO-1 and CT45). For MAGEA, NY-ESO-1, CT7, CT10 and CT45, the frequency of expression in all ER-negative (and either HER2+ or HER2-) tumors was 24.5%, 17.7%, 19.2%, 11.7% and 15.8%, respectively. In comparison, only 2.1% to 6.3% of ER-positive tumors expressed these CT antigens. The frequencies of CT expression in the triple-negative (ER-HER2-) carcinomas are similar to the ER-negative group, being 24.0%, 19.1%, 14.2%, 10.2% and 18.2% for MAGEA, NY-ESO-1, CT7, CT10 and CT45, respectively.

### ER-negative tumors often co-expressed multiple CT antigens

**Figure 5:**
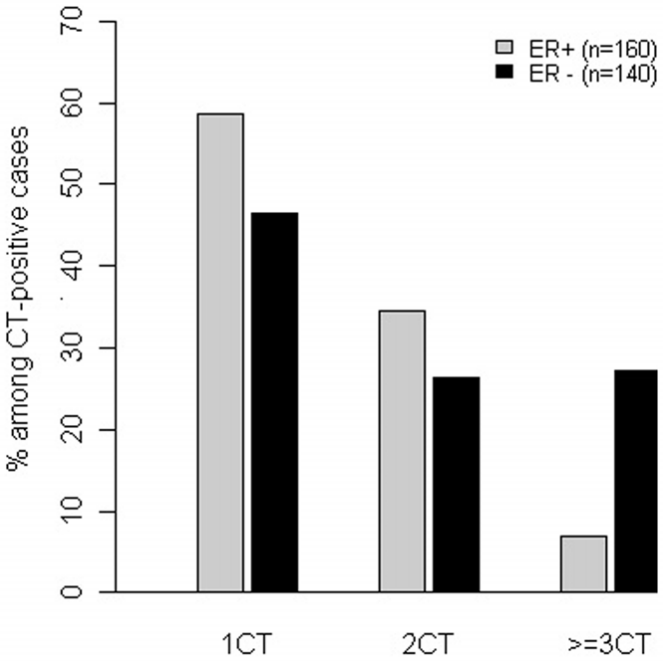
Distribution of cases expressing one, two or multiple CT antigens among all CT–positive cases in percentage. Significantly greater proportion of ER negative tumors expressed ≥3 antigens when compared to ER+ tumors (P = 0.026).

Breast cancer specimens positive for any one CT antigen were found to be often positive for additional CT antigens, and this phenomenon was particularly striking in ER-negative tumors (Figure 5). Of 265 ER-negative cases, 125 (47.2%) expressed at least one CT antigen, in contrast to 29 of 189 (15.3%) of ER-positive cases. Among these 125 CT-positive cases, 34 (27.2%) expressed three or more of the eight CT antigens examined. In comparison, 27 of 29 CT-positive ER-positive cases expressed only one or two CT antigens, and only 2 (6.9%) in the ER-positive group, significantly less frequent than the ER-negative group (*p = *0.026).

### HER2-positive versus HER2-negative tumors

**Figure 6:**
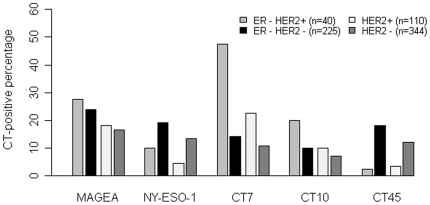
Distribution of CT antigen expression percentages in HER2 positive and HER2 negative breast cancer. Comparisons were carried out for each CT antigen between the ER-HER2+ and the ER-HER2- (triple-negative) cases and between all HER2+ and HER2- cases. Significant differences were found for both comparisons for CT7 and CT45 only (P≤0.01), with CT7 more frequently expressed in HER2-positive tumors, whereas CT45 more frequently in HER2-negative tumors.

CT expression in HER2-positive versus HER2-negative breast cancers was compared between the ER-HER2+ and ER-HER2− groups to exclude potential influence of ER status, and all HER2+ and all HER2− cases were also compared in parallel (Figure 6). Of the 265 ER-negative tumors, 40 (15%) were HER2−positive. ER−HER2+ and ER−HER2− showed similar expression frequency of MAGEA, NY-ESO-1 and CT10 (*p = *0.69, 0.26 and 0.11, respectively). In contrast, CT7 showed more frequent expression in the ER−HER2+ group (47.5% versus 14.2%, *p<*0.001), and CT45 were more frequently expressed in the ER-HER2− group (2.5% versus 18.2%, *p = *0.009). These differences in CT7 and CT45 expression remained when all HER2 positive cases were compared to all HER2 negative cases (*p = *0.002 and 0.02 for CT7 and CT45, respectively).

### Correlation with other pathologic parameters

Correlation between CT expression and nuclear grade, tumor size and nodal status was evaluated in the Cornell series irrespective of the ER and HER2 status (Table 2). Breast cancers with high nuclear grade showed significantly more frequent CT expression than those with low and intermediate nuclear grades for all five main CT antigens (p≤0.003). Tumors equal to or greater than 2 cm in size (pT2) also expressed MAGEA, CT7 and CT10 at higher frequency than tumors of smaller size (p = 0.003, <0.001, and  = 0.01, respectively). However, no difference was seen between tumors 1–2 cm in size and those that were 1 cm or less. Lymph node status did not appear to affect CT expression frequency (lymph node positive versus negative, p = 0.17 to 0.83).

**Table 2:** Correlations between CT expression and clinicopathological parameters.

	MAGEA	NY-ESO-1	CT7	CT10	CT45
**Nuclear grade**					
1–2	6/141(4.3%)	5/141(3.5%)	7/141(5.0%)	6/141(4.3%)	5/141(3.5%)
3	42/146(28.8%)	20/146(13.7%)	37/146(25.3%)	19/146(13.0%)	21/146(14.4%)
**P Value**	<0.001	0.003	<0.001	0.01	0.002
*Tumor size*					
≤2 cm	22/193(11.3%)	13/193(6.7%)	17/193(8.8%)	10/193(5.2%)	17/193(8.8%)
>2 cm	24/93(26%)	11/93(11.8%)	26/93(28.0%)	14/93(15.1%)	8/93(8.6%)
**P Value**	0.003	0.17	<0.001	0.01	1
**Lymph node**					
Positive	12/98(12.2%)	7/98(7.1%)	18/98(18.4%)	7/98(7.1%)	8/98(8.2%)
Negative	32/167(19.2%)	15/167(9.0%)	24/167(14.4%)	14/167(8.4%)	16/167(9.6%)
**P Value**	0.172	0.653	0.39	0.82	0.83

## Discussion

Invasive ductal carcinoma of the breast is a biologically and clinically heterogeneous group of carcinomas. While surgical resection is the main treatment for early disease, adjuvant systemic treatments are required or recommended in patients with visceral metastasis, node-positivity, or high-risk node-negative disease, the last group including all ER-negative carcinoma (NCI treatment guideline http://www.cancer.gov/cancertopics/treatment/breast). Hormonal therapy alone or hormonal therapy plus chemotherapy is the usual treatment of choice for ER-positive tumors. For most patients with ER-negative carcinoma, however, chemotherapy is the main treatment option. An exception to this scheme is the ER-negative, HER2-positive subgroup, for which anti-HER2 monoclonal antibody is often effective, and this has been used in the standard care of metastatic HER2-positive breast cancer. Despite these therapeutic modalities, all ER-negative breast cancers, including the HER2-positive cases, carry a much poorer prognosis than ER-positive tumors [18], and additional treatment options are highly desirable and continuously sought for, particularly for the subgroup of ER, PR, and HER2 triple-negative carcinoma.

In our previous study [12], we analyzed mRNA transcripts in breast cancer cell lines and cancer specimens using data sets derived from massively parallel signature sequencing (MPSS) and publicly available expression microarray data. These analyses identified more frequent CT expression in estrogen and progesterone receptor negative breast cancer, including NY-ESO-1, LAGE-1, MAGEA, PAGE4 and SSX1. Immunohistochemical analysis, performed for NY-ESO-1 and MAGEA, confirmed the finding at the protein level and also indicated that triple-negative breast cancer, in particular, might be a “CT-rich” tumor type. Our present study extends the analysis to encompass eight CT-X antigens—MAGEA, NY-ESO-1, CT7, CT10, CT45, GAGE, NXF2 and SAGE1, and showed higher expression rates in ER negative tumors for all CT antigens. HER2-negative status, however, does not appear to further increase the CT expression except in CT45. On the other hand, CT7 expression was found to be more frequent in the HER2-positive tumor. The reason for this difference in CT7 and CT45 is unclear and probably should be interpreted with caution, as the number of positive cases is relatively small in some groups.

We found different CT antigens to be significantly different in their CT expression rate in breast cancer, and GAGE, NXF2 and SAGE1 were rarely expressed even in the ER-negative group. This finding is similar to our RNA expression data in lung cancer. In contrast, GAGE mRNA and protein was expressed in much higher frequency in malignant melanoma (unpublished data). The remaining five CT antigens that were studied–MAGEA, NY-ESO-1, CT7, CT10 and CT45, were expressed in up to 25% of ER-negative tumors individually, and 47% of ER-negative cancer expressed at least one CT antigen. This moderate frequency of expression, in conjunction with the previously demonstrated spontaneous immuogenicity of these five antigens in cancer patients [19], [20], [21], [22], suggest this group of CT antigens as potential cancer vaccines targets for ER-negative breast cancer, including the triple-negative subgroup. The fact that ER-negative carcinomas often showed co-expression of multiple CT antigen indeed would further suggest polyvalent CT vaccine as an approach that should be explored.

The frequency of CT antigen expression in breast cancer has only been examined in a few previous studies, and the reported data were highly variable [11], [12], [13], [14], [15]. By RT-PCR, Sugita et al. [14] identified NY-ESO-1 mRNA was in 42% of breast cancer, in comparison to a 13% expression rate reported by Mischo et al. [13]. However, many of the NY-ESO-1 positive cases in the series of Sugita et al. contained only low levels of NY-ESO-1 mRNA, and the authors could only detect NY-ESO-1 protein expression in a single positive case by immunohistochemistry. Theurillat et al. [15] studied a largest series of 1355 breast cancers by immunohistochemical analysis in a TMA format, and they similarly observed a low rate of NY-ESO-1 protein expression, shown in only 2.1% (28/1355) of the cases. This, however, is in sharp contrast to Bandic et al. [11] who described a 40% NY-ESO-1 positive rate in their study of recurrent ductal breast cancer, and a high 74% MAGE-A4 positivity was also described in the same study. Several technical variations could possibly contribute to this wide variation in the observed CT expression frequency. One was the use of TMA in the current and some previous studies [12], [15] versus whole tissue sections in others [11], [13], [14]. Since CT expression in cancer is often heterogeneous, TMA-based analysis is likely to have a higher false-negative rate due to sampling errors, and the frequencies obtained by TMA analysis could be lower for this reason. Other factors that differed among different studies included the use of different antibodies and antigen-retrieval techniques. For MAGEA expression, two monoclonal antibodies were used in these studies, 57B [11], [12], [13], [14], [15] and 6C1 [12], and we have chosen to use 6C1 for our study as it has been shown to recognize MAGE-A1, -A2, -A3, -A4, -A6, -A10 and -A12, whereas 57B does not recognize MAGE-A10 [23] and is not commercially available. In addition to these technical differences, we believe that a major source of variation is the different case distribution of ER-positive versus ER-negative breast cancers in these studies. Since more than 80% of the breast cancers are ER-positive, a collection of unselected breast cancers would consist mainly of ER-positive tumors. This would explain the low (2.1%) NY-ESO-1 positive rate in the large series of Theurillat et al. [15] which is close to the 2.4% expression rate in the ER-positive group of our Cornell series. In comparison, 65% of the cases (53/81) studied by Bandic et al. were ER-negative tumors, and this would at least partially account for the higher expression of NY-ESO-1 and MAGE-A4 proteins in their series of recurrent breast cancer. However, our 17.7% NY-ESO-1 positivity and 24.5% MAGEA positivity in the ER-negative group were still substantially lower than the 40% and 70% positivity rate that they reported, suggesting that other factors likely exist. For instance, recurrent breast cancer may also be of higher histological grade in general, which we also found to correlate with increased CT expression. If confirmed, it would suggest a possible role for CT-based cancer vaccine in the treatment of recurrent, both ER+ and ER-, breast cancers.

Except for CT7, all CT antigens analyzed in this study showed nuclear and/or cytoplasmic staining patterns identical to their expression in the testicular germ cells, i.e. MAGEA, NY-ESO-1 and GAGE were present in both nuclear and cytoplasmic compartments, and CT10, CT45, NXF2 and SAGE1 were purely nuclear proteins. However, CT7, normally expressed in the cytoplasm of spermatogonia, was noted to be both nuclear and cytoplasmic in most tumor cells. This has been previously reported by Tinguely et al. in multiple myeloma [24]. Intriguingly, myeloma patients with only cytoplasmic CT7 expression were found to have better prognosis than those with mixed nuclear and cytoplasmic expression, leading the authors to speculate that nuclear CT7 expression might lead to proliferative or survival advantages of these neoplastic cells. Whether this differential subcellular localization has a prognostic influence in breast cancer is unknown, but no apparent correlation was seen to the ER and HER2 status, or to the nuclear grade, nodal status or tumor size (data not shown). In addition to this aberrant nuclear expression of CT7, MAGEA and NY-ESO-1 also showed significant difference in their subcellular distribution. Since MAGEA is a multigene family of more than 10 genes, at least 7 of them recognized by the antibody used, it is likely that some of the difference of nuclear versus cytoplasmic staining might be resulted from the expression of different MAGEA gene(s). However, this explanation cannot account for the difference observed in NY-ESO-1 staining, and the possible biological basis and consequence of nuclear versus cytoplasmic NY-ESO-1 expression are unclear at present.

In summary, prior literature suggested breast cancer as a relatively “CT-poor” tumor type, and CT antigen-based therapeutic cancer vaccine trials, e.g. MAGEA3 and NY-ESO-1 trials, have not been actively pursued in breast cancer for this reason. Our present study demonstrated higher expression frequencies of MAGEA, NY-ESO-1 and other CT antigens in the ER-negative group, including the ER/PR/HER2 triple-negative subgroup, of invasive ductal carcinoma. Considering the limited treatment options and the poor prognosis of the triple-negative breast cancer, further investigations to explore the potential of CT antigen-based immunotherapy in this patient group is clearly warranted.

## Materials and Methods

### Ethics Statement

This study was conducted according to the principles expressed in the Declaration of Helsinki and followed the protocols approved by the institutional review boards (IRB) of Weill Cornell Medical College and University of California-San Francisco.

### Tissues and tissue microarrays

Formalin-fixed paraffin-embedded breast cancer specimens used for this study were procured from the Department of Pathology and Laboratory Medicine at New York Presbyterian Hospital-Weill Cornell Medical Center and the Department of Pathology at the UCSF Medical Center, following protocols approved by the IRB of the two institutions.

For the Cornell series, pathology reports from 2006 to 2009 were searched for cases with the diagnosis of invasive ductal carcinoma. The ER and HER2 status, available as standard diagnostic work-up, was recorded. For the purpose of this study, ER-positivity was defined as at least moderate nuclear ER staining in more than 10% of tumor cells, and HER2 positivity was documented by either 3+ immunohistochemical staining with anti-HER2 antibody in >30% of tumor cells, or by a positive fluorescent in-situ hybridization assay, defined as >2.2 HER2 gene copies per tumor cell. Hematoxylin and eosin stained slides were reviewed and a representative block was retrieved from each case for the construction of tissue microarray (TMA). Final case list selected for TMA comprised 289 invasive ductal carcinomas, including 163 ER-positive (54 ER+HER2+, 109 ER+HER2-) and 126 ER-negative specimens (27 ER-HER2+, 99 ER-HER2−). All ER-HER2− cases were also PR-negative, representing so-called “triple negative” breast cancer. The tumor size, lymph node status and nuclear grade were also recorded for all cases. The UCSF series, identified in a similar fashion from archival materials of 1998–2008, was obtained to specifically expand the categories of ER/PR/HER2 triple-negative cases and HER2+ cases. The UCSF series comprises 165 cases, including 139 ER-negative (13 ER-HER2−, 126 ER-HER2−) and 26 ER-positive (16 ER+HER2+, 10 ER+HER2−) specimens. For the immunohistochemical analysis, both Cornell series and the UCSF series were constructed into TMAs with 0.6 mm and 1.5 mm tissue cores, respectively. In total, 454 breast cancers were evaluated in this study, including 225 ER/PR/HER2 triple-negative, 119 ER+HER2−, 70 ER+HER2+, and 40 ER-HER2+ ductal breast carciniomas.

### Monoclonal and polyclonal antibodies

The antibodies used are summarized in Table 3. Antibodies against GAGE, SAGE1 and MAGE-A were purchased commercially. GAGE antibody, produced against GAGE-7, is expected to react with all GAGE gene products due to the extreme high sequence homology among the GAGE proteins. MAGEA monoclonal antibody 6C1, produced against MAGE-A1, has been shown to be broad-reactive for gene products of MAGEA multigene family, including MAGE-A1, A2, A3, A4, A6, A10 and A12 protein [23].

**Table 3:** Antibodies used in the present study.

Antigen	Origin	Code	Dilution	Source
MAGEA	Mouse	6C1	1 µg/ml	Santa-Cruz Biotech, Santa Cruz, CA
NY-ESO-1	Mouse	E978	1 µg/ml	*
CT7	Mouse	CT7-33	0.1 µg/ml	*
CT10	Mouse	LX-CT10-5	3 µg/ml	*
CT45	Mouse	LX-CT45-10	1∶5000 (ascites)	*
GAGE	Mouse	clone 26	0.1 µg/ml	BD Biosciences, San Jose, CA
NXF2	Mouse	LX-NXF2-1	1∶500 (ascites)	*
SAGE1	Rabbit	polyclonal	1.5 µg/ml	Sigma-Aldrich, St. Louis, MO

Research antibodies produced in our laboratory.

Antibodies against the other CT-X antigens NY-ESO-1, CT7, CT10, CT45 and NXF2 were produced and characterized in our laboratory. Antibodies against NY-ESO-1, CT7, CT10 and CT45 have been previously described [25], [26], [27], [28]. For generating NXF2 monoclonal antibodies, full-length NXF2 cDNA sequence was cloned into prokaryotic expression vector pQE30 (Qiagen), and subsequent induction of recombinant protein synthesis and purification by Ni^+2^ affinity chromatography were performed as previously described [29]. Mouse monoclonal antibodies were then produced and characterized following previously described protocols [25]. The specificity of the anti-NXF2 monoclonal antibodies was confirmed by ELISA and by positive immunohistochemical staining of spermatogonia in testis and negative staining using a panel of normal adult tissues (data not shown).

### Immunohistochemical analysis

Immunohistochemical (IHC) analysis was performed on formalin-fixed paraffin-embedded tissues. Five mm sections of TMA on coated slides were deparaffinized, rehydrated and treated in H_2_O_2_ to block the endogenous peroxidase activity. The sections were then subjected to antigen retrieval by autoclaving for 15 minutes in 10 mM citrate buffer, pH 6.0. The sections were incubated with the primary antibody for one hour at room temperature, followed by detection using DAKO Envision+ horseradish peroxidase mouse (or rabbit) detection system (DakoCytomation) and DAB as the chromogen. The slides were counterstained with hematoxylin and evaluated. Any staining on cancer cells is regarded as positive, and the immunoreactive intensity was recorded as + to +++ (and a numeric score of 1 to 3). The heterogeneity of staining was also recorded as very focal (<10% of tumor cells), focal (10–50%) and diffuse (>50%), and a corresponding numeric score of 1 to 3 was assigned. The two scores were combined, given any positive case a score of 2 to 6. Three cores of normal adult testis were included in each TMA block in the Cornell series and served as the positive control.

### Statistical analysis

Differences in the frequency of CT antigen expression in different groups were examined using Fisher's exact test. Multivariable logistic regression was used to adjust for potential differences in data source. For Cornell series where the other clinical parameters were recorded, association between CT antigen expression and each of the clinical parameters were examined using Fisher's exact test.
